# Diet Quality of Malaysians across Lifespan: A Scoping Review of Evidence in a Multi-Ethnic Population

**DOI:** 10.3390/nu13041380

**Published:** 2021-04-20

**Authors:** Amutha Ramadas, Su Ming Tham, Shehzeen Alnoor Lalani, Sangeetha Shyam

**Affiliations:** 1 Jeffrey Cheah School of Medicine and Health Sciences, Monash University Malaysia, Jalan Lagoon Selatan, Bandar Sunway 47500, Malaysia; amutha.ramadas@monash.edu (A.R.); suming.tham@gmail.com (S.M.T.); 2 School of Medicine, International Medical University, Jalan Jalil Perkasa 19, Bukit Jalil, Kuala Lumpur 57000, Malaysia; shehzeen_lalani@yahoo.com; 3 Division of Nutrition and Dietetics, School of Health Sciences, International Medical University, Jalan Jalil Perkasa 19, Bukit Jalil, Kuala Lumpur 57000, Malaysia; 4 Centre for Translational Research, IMU Institute for Research and Development (IRDI), International Medical University, Jalan Jalil Perkasa 19, Bukit Jalil, Kuala Lumpur 57000, Malaysia

**Keywords:** diet quality, diet index, diet variety, nutrition assessment, Malaysia

## Abstract

Malaysia is a rapidly developing economy experiencing a nutrition transition. It suffers from a double burden of over- and undernutrition, making it essential to understand diet quality in the population. In this scoping review, we have collated the existing literature on Malaysian diet quality, including factors that influence it, and the association between diet quality and health outcomes across the lifespan of Malaysians. Overall, diet quality was poor in all age groups studied. The Healthy Eating Index (HEI) and its iterations were predominantly used in urban and clinical settings to evaluate diet-chronic disease relationships. These indices were significantly associated with cardio-metabolic and disease risks in adults. The Diet Diversity Score (DDS) and Food Variety Score (FVS) were used to gauge diet quality in maternal and child nutrition studies and were associated with appropriate growth and caloric intake. Deficiencies were found in fruit, vegetable, legumes, and dairy intake. Meat, salt, and sugar intake were found to be excessive in many studies. The findings can inform policies to improve diet quality in this population. The review also identified knowledge gaps that require further investigation.

## 1. Introduction

Malaysia is an upper-middle-income country and is an open economy with a high trade-to-gross-domestic-product (GDP) ratio [1]. As Malaysia converges with advanced economies, challenges remain in achieving equitable education, health, nutrition, and social protection outcomes [1]. Rapid development and globalization have also spurred a nutritional transition with an overall shift away from the traditional and more globalized dietary patterns [2,3]. This dietary shift is paralleled by the double burden of obesity and undernutrition, with the former being more generalized in prevalence across the nation and the latter concentrating in segments of under-developed and low-income areas, especially among children [4]. The scale of the prevalence of overweight status and obesity, which has risen unabated over the last two decades, with no clear urban-rural divide, is a cause for national concern [3,5]. This public health concern has prompted the development of the National Strategic Plan for Non-communicable Diseases (2015–2025) with specific targets to halt obesity and diabetes [6]. In addition to this, stunting in children is still a matter of concern in Malaysia [4,7]. Among the key priority areas identified for the development of human capital in Malaysia are nutritional interventions to reduce childhood stunting and adequate social welfare protection [1,2]. Thus, evaluating diet quality using a life course approach is of prime importance in this rapidly transitioning society to understand and optimize the population’s diet.

Nutrition research aims to optimize diets to promote health and prevent diseases [8,9], as poor diet quality is globally recognized as a crucial, yet modifiable risk of adverse health outcomes [10]. Diet quality refers to the extent to which an individual’s diet conforms to the principles of being balanced, healthy, and diversified and provides the essential nutrients needed for a healthy life [11,12,13]. Diet quality functions as a risk assessment tool to predict outcomes from various chronic diseases, including cardiovascular diseases, diabetes, obesity, and cancer risk, both in children and adults [11,14,15]. Practical and valid metrics support efforts to elucidate associations between diet and health outcomes and utilize the understanding to intervene and optimize health to measure diet quality [11].

Diet quality is challenging to quantify [14] and involves evaluating one or more of the following three domains, depending on the objectives and context of usage: (a) nutrient adequacy, (b) food variety and diversity, and (c) moderation of food groups and nutrients [16]. Nutrient adequacy refers to the consumption of appropriate amounts of energy and all essential macro- and micronutrients suited to a person’s age, sex, and health status. Diversity refers to the variety of food groups included in a diet. Finally, moderation refers to consuming food groups within their limits, as an excess may be unhealthy [17]. Implicit in this measurement is that diet quality defines the agreement between individual eating behavior and healthy eating as defined by the existing national dietary guidelines [12,13]. The subtle variations in the measurement of diet quality indicators suggest that diet quality metrics/indices are best customized to a population depending on their nutritional requirements, cultural acceptability, and the population’s trajectory in the nutritional transition. This poses an additional challenge to countries like Malaysia that have to be mindful of the existing issue of undernutrition while the prevalence of over-nutrition and its consequences continues to grow.

Several diet quality metrics that vary both in their foundation and dietary components evaluated are used in nutrition research. These fall under three major categories: (a) nutrient-based indicators, (b) food/food group-based diversity indicators, and (c) combination indexes [12,18]. Metrics that fall into each of these categories are shown in Table 1.

The four most globally used and extensively validated indices are the Healthy Eating Index (HEI), the Healthy Diet Indicator (HDI), the Diet Quality Index (DQI), and the Mediterranean Diet Score (MDS) [12] and their adaptations. Some of these scores are tied to national dietary guidelines and therefore are specific to the country guidelines they are calibrated to. The HEI assesses how well a diet aligns with the key recommendations of Dietary Guidelines for Americans [19]. McCullough et al. [20] created an alternative to the HEI to improve the original algorithm. This 9-component Alternate Healthy Eating Index (AHEI) focuses on foods and nutrients, and the prevention of chronic disease risks [12]. These indices have been adapted to suit country-specific dietary guidelines, and a modified version of the HEI is available for Malaysia [21,22]. Diet quality indices have also been specifically developed and validated for Malaysia, based on the Malaysian Dietary Guidelines [23].

Some dietary scores facilitate cross-country comparisons and therefore are intended to be used without modifying the items or scoring across countries. Globally calibrated diet quality indices include the Healthy Diet Indicator (HDI), the Diet Quality Indicator International (DQI-I), the Food Variety Score (FVS), the Diet Diversity Score (DDS), and the Mediterranean Diet Score (MDS). The HDI is used worldwide and is based on adherence to the World Health Organization’s (WHO) nutrition guidelines, while the DQI-I was created in 2003 to enable cross-cultural diet quality comparisons [24]. The MDS comprises 11 components and is based on epidemiological findings of a beneficial diet on cardiovascular health [25,26]. However, in low-resource settings with undernutrition, the FVS and DDS are more straightforward measures that can be used without the need for a food composition database [27,28]. The FVS is defined as the count of food items consumed over 24 h, from a possible total of 45 items [27]. The DDS is used to measure the diversity within and between food groups by a simple food group count [28]. Thus, the FVS and DDS can be quick screeners of diet quality in populations at risk for undernutrition.

Malaysia has carried out several studies on diet quality and its health effects across various age groups and settings. These efforts remain disconnected, and an evaluation of what is known and what remains to be studied will be vital to support future work in the area. Thus, we have reviewed the existing work done within Malaysia, documenting diet quality across the life course, factors associated with diet quality, and their impact on health outcomes.

## 2. Materials and Methods

### 2.1. Study Design

This scoping review was undertaken based on the methodology outlined by Arksey and O’Malley et al. and the Joanna Briggs Institute [29,30,31]. The protocol adopted for this scoping review was based on the Preferred Reporting Items for Systematic Reviews and Meta-Analyses (PRISMA) Extension for Scoping Reviews [32]. Since the data included in this review were available in the public domain, ethical approval was not sought.

In this review, we have defined diet quality as an indicator of the extent to which a diet conforms to (i) general healthy eating principles of balance, variety, and moderation and (ii) recommended nutrient intake, including suggested limits to negative nutrients such as fat and salt intakes. The working definition of diet quality for this review specifically looks at the alignment of the diet with these principles as enshrined in the Malaysian Dietary Guidelines (1999 or 2010) [33,34] and Malaysian Recommended Nutrient Intake (2005, 2017) [35,36] that were current at the time the individual studies were conducted. We therefore included studies that used food/food group-based diversity indicators or food combination indices to evaluate dietary quality.

In this scoping review, we have included both factors that affect the diet quality of multi-ethnic Malaysians and the association between diet quality and health outcomes across the lifespan of Malaysians.

### 2.2. Search Strategy

Peer-reviewed scientific publications were searched in Ovid MEDLINE, PubMed, Embase, Scopus, ScienceDirect, and Ebscohost databases up to 31 December 2020.

A search strategy was devised using the keywords and Boolean operators (‘diet quality’ OR ‘diet quality index’ OR ‘healthy eating index’ OR ‘food variety’ OR ‘diet diversity’ OR ‘diet diversity score’) AND (Malaysia). Supplementary Table S1 shows the search syntax and strategy for Ovid MEDLINE conducted on 28 January 2021. The syntax and strategy were adapted for the remaining electronic databases.

### 2.3. Study Selection

Covidence software was used to facilitate the study selection process [37]. Records yielded from the database searches were imported to Covidence. Duplicate records were removed before titles and abstracts of articles were screened to gauge study eligibility. In the final step, full-text articles were reviewed in accordance with the review’s inclusion and exclusion criteria. Screening of all studies was independently conducted by two researchers (S.A.L. and S.M.T.) at both stages. We also performed a manual search of the references listed in the included studies. A third researcher (A.R.) resolved conflicts in agreement, if any, between S.A.L. and S.M.T. Pre-determined eligibility criteria were used to screen for peer-reviewed articles that had explored any aspects of diet quality (Supplementary Table S2).

### 2.4. Data Extraction

A two-step approach was employed to conduct the data extraction. First, we developed a Google Sheet-based template to extract key data of interest—study design, objective, population studied, sample size, setting, diet quality measures used, and main findings. Following that, the studies were reorganized according to factors of interest: (i) setting or population, (ii) diet quality measures used, (iii) factors influencing diet quality, and (iv) health outcomes associated with diet quality.

## 3. Results

### 3.1. Study Selection and Characteristics

**Figure 1:**
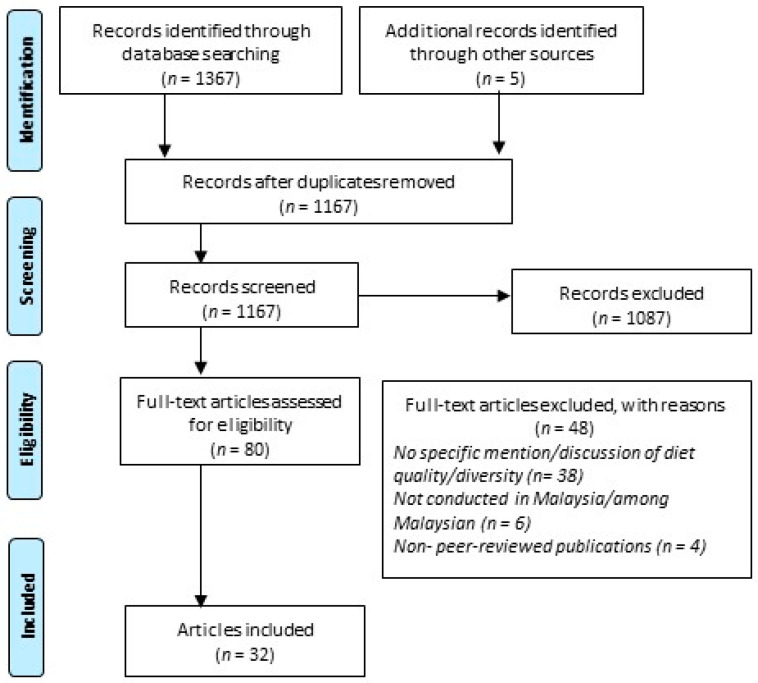
Preferred Reporting Items for Systematic Reviews and Meta-Analyses (PRISMA) flow chart.

We identified 1367 records in total through database searches. An additional 5 records were obtained from manual searches. We subsequently removed 200 duplicate records. The titles and abstracts of the remaining 1167 records were screened for inclusion. Following that, we reviewed the full texts of 80 articles for eligibility. Finally, 32 articles representing 29 unique studies were included in the review (Supplementary Table S3). The search and selection of the studies are summarized in Figure 1.

### 3.2. Study Settings and Populations

**Figure 2:**
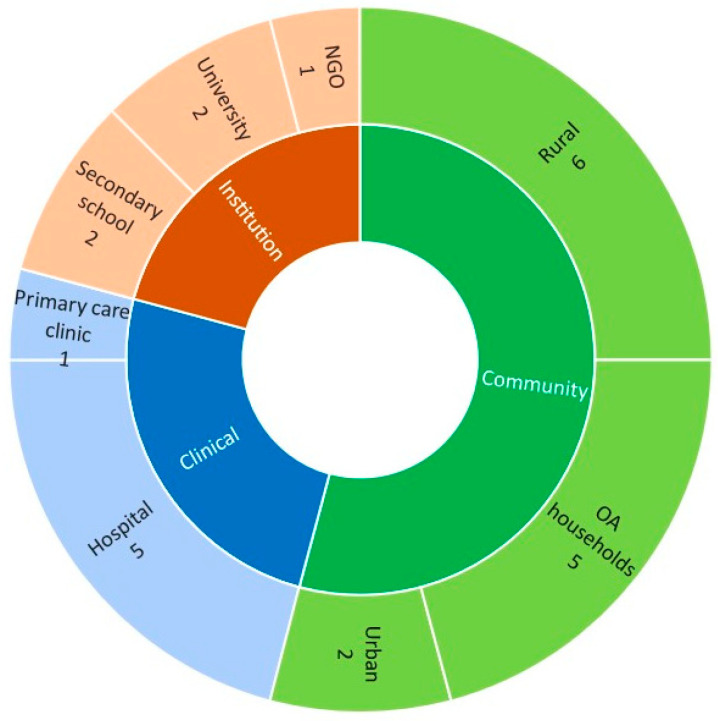
Distribution of the studies according to study setting or location. Note: OA = Orang Asli; NGO = Non-governmental organization.

The majority of the studies on diet quality were conducted in community settings, with 6 studies in rural settings, followed by 5 in Orang Asli (OA) households (Figure 2). Interestingly only 1 study was conducted among urban community dwellings. Hospitals (*n* = 5) were a more familiar setting for subject requirements than primary care clinics (*n* = 1). Five studies were conducted in organizations or institutions.

Thirteen studies were conducted in a multi-ethnic population, and 6 were explicitly done among adults (Table 2). Surprisingly, only 1 study of multi-ethnic children was reported, although 2 studies of OA children were conducted. Four studies investigated mothers with at least 1 child in the Malay, Indian, and OA populations. Adults with diabetes or prediabetes were the subjects of investigation in 3 studies, followed by studies of adults with breast cancer (*n* = 2).

### 3.3. Diet Quality Measures and Status

**Figure 3:**
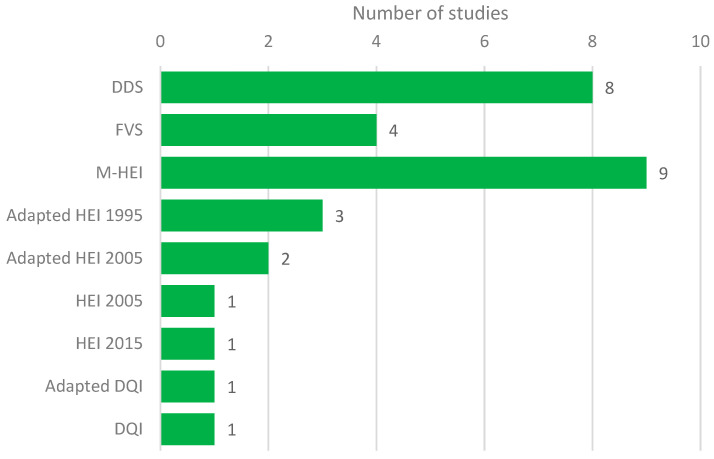
Distribution of the studies according to diet quality measures used.

The Malaysian Healthy Eating Index (M-HEI) was the most commonly used diet quality measure (*n* = 9) in Malaysia (Figure 3), followed by the DDS (*n* = 8) and FVS (*n* = 4). A detailed description of the measures is presented in Supplementary Table S4.

The diet quality of rural children was poor with a poor score for vegetables, fruits, dairy, and overall food variety [38]. More than two-thirds of OA children (73.8% of males and 59.1% of females) had poor diet quality [56]. Poor diet quality among households with individual food insecurity, child hunger, and household insecurity measured 81%, 77.8%, and 63.8%, respectively. Diet quality remained poor in OA children a decade later, with 92.1% in the middle tertiles of the DDS or lower [57]. 

A study of the urban Malaysian community showed the paradox of a high prevalence of low-medium FVS (67.7%) concurrently with a high DDS (88.4%), likely due to households consistently purchasing familiar yet cheaper foods [43]. The diet quality of adult urban Malaysians was found to need improvement (average M-HEI score: 61.3 ± 10.9) [44]. Interestingly, while a trial reported higher diet quality with the intervention meal, the group remained in the “needs improvement category” [66], and a similar finding was reported earlier in another intervention conducted among adult men [45]. Karppaya and colleagues [58] reported a mean DDS score of 9.5 ± 4.2 in an OA community, with similar scores between genders.

More than 55% of urban women were found to have diet quality that “needs improvement”, with Indian women having the lowest HEI score (75.7 ± 8.1) among the ethnic groups [46], while OA women were found to have poor diet quality and an even lower score, of 45.3 ± 7.5 [21,22]. However, diet quality status in adult women was associated with food security status rather than ethnicity alone in several other studies. Food-secure women have been reported to have higher diet quality, assessed with the DDS and M-HEI, compared to women experiencing food insecurity and child hunger [21,22,47,50,55]. The association between food security and diet quality was also documented in women with children [51,52,53,67]. The only study that reported on pregnant women found lower M-HEI scores throughout their pregnancy (ranging between 52.7–56.1%) [54].

Surprisingly, older adults (M-HEI score: 70.2 ± 12.0) had significantly better diet quality than the younger age group (59.7 ± 9.9) [44]. This finding appears to be consistent with a recent study reporting an M-HEI score of 66.9 ± 9.9 in women above 50 [48]. Nohan et al. [49] also reported that almost 75% of the older adults in their study had good diet quality.

Diet quality assessments in specific groups also have been reported in the past. A recent study reported that a significant proportion of intellectually disabled men (60%) were required to improve their dietary intake, and none had good diet quality [63]. Ng et al. [64] reported a mean total HEI score of 63.9 ± 8.8 in women with breast cancer. This was consistent with an earlier study that found a total HEI score of 64.8 ± 9.7 and 64.3 ± 9.3 among pre-and postmenopausal breast cancer cases, respectively [65]. 

Tiew and colleagues [60] reported a mean Food Group Score (FGS) of 4.1 ± 0.8 and Serving Score (SS) of 12.8 ± 3.5 among adults with diabetes. Interestingly, while this study found that one-third of the patients had a perfect FGS, only 1.8% were identified with perfect SS. The majority of people with diabetes (76.8%) needed to improve their diet quality [61]. A similar finding was reported by Siddiqui et al. [62], where diabetic patients achieved a total M-HEI score of 58.1 ± 9.1. However, pre-diabetic individuals in this study may have been at greater risk of having poor diet quality as this group only achieved a composite M-HEI score of 55.9 ± 7.2.

### 3.4. Demographic and Nutritional Factors Associated with Diet Quality

Higher or better education [49,60], maternal years of schooling [57], being a female [39], not working [60], and higher household and personal income [60] have been consistently associated with better diet quality among adults and older adults (Table 3). Negative associations were found between diet quality and being a male [40] or member of the non-Jah Hut OA subtribe [57]. However, the associations between age [43,44,49,57] and Malay ethnicity [40,46,49] with diet quality have been inconsistent. 

Food insecurity [21,22,47,50,51,52,53,55,67] has been consistently associated with poor diet quality in adults (Table 4). Limited evidence was found for positive associations between diet quality and household food expenditure [43], higher energy-adjusted daily dietary cost [44], healthy lunch meals [66], availability of healthy foods [40], self-efficacy of healthy eating [40], energy intake [59], and higher carbohydrate intake [44]. Negative associations were found between diet quality and nutritional knowledge [41], eating out [46], food neophobia [41], sensory motives [41], and higher fat intake [21,22,44].

### 3.5. Associations between Diet Quality, Anthropometry, and Clinical Outcomes

Limited studies investigated the association between diet quality and health outcomes. Negative associations between diet quality and higher BMI/obesity [42,60,64], abdominal obesity [49,60], poor body composition [49], and mid-upper arm circumference (MUAC) [49] have been reported among adults and older adults (Table 5). Positive associations between diet quality and skeletal muscle and handgrip strength [49] were observed among older adults. Saibul et al. [59] reported the only study investigating the dual burden of malnutrition and found a positive association with diet quality in mothers but a negative association in children. None of these associations were explored in adolescents or specifically in adult men. 

None of the reported studies explored the impact of diet quality on clinical outcomes in children, adolescents, young adults, or adult men (Table 6). Limited evidence pointed toward positive associations between good diet quality and higher serum hemoglobin [64] and excessive gestational weight gain [54] in women. A negative association between diet quality and breast cancer risk was reported by one study [54]. Intriguing positive associations between diet quality and hypertension among older adults [49], postprandial glucose level among prediabetic individuals [62], and insulin treatment among diabetic patients [60] were found.

## 4. Discussion

In this scoping review, we collated the existing literature to understand what is known about the Malaysian population’s diet quality. We reviewed findings that indicate the quality of Malaysian diets, factors that influence them, and the impact of diet quality on health outcomes across the lifespan of Malaysians. The findings of this review, we believe, will provide an actionable reference for policymakers and researchers to tackle the double burden of under- and over-nutrition in Malaysia, a rapidly growing middle-income country.

The choice of diet quality indicators used in Malaysian studies reflects the co-existence of over- and undernutrition. Food variety-based scores are more useful in determining diet quality in maternal and child nutrition, while the HEI and DQI are more useful in studying the relationships between diet and non-communicable disease (NCD) risk [10]. Accordingly, Malaysian diet quality studies focusing on nutrient adequacy in maternal and child nutrition used the FVS or DDS, while studies focusing on NCD risks used the HEI or its modified versions. Defined by the nature of scoring, food variety-based scores were associated with higher energy intake in the study of mother and child dyads whereas higher scores on the HEI or its modified versions were associated with lower fat (higher carbohydrate) intake in the studies included in this review. The high prevalence of obesity and related chronic diseases relative to undernutrition could explain the predominant use of the HEI or DQI and their derivatives in Malaysian diet quality studies. It is therefore interesting to see that studies conducted among indigenous communities [57,58], rural settings [50,51,52,53,55,67], and in lower-income neighborhoods [47] used the DDS or FVS. These studies also tended to focus more on sociodemographic factors affecting food insecurity and the association of diet quality indices with nutritional status. Studies using the HEI or DQI or their modified versions tended to be more in the urban [39,44,45,46,48,49,63,66] and clinical settings [54,60,61,62,64,65]. The latter were also more likely to focus on the association of diet quality with cardiometabolic or chronic disease risks. However, there were exceptions to this assumption. Chua et al. [38] and Chong et al. [21,22] used the HEI versions to evaluate diet quality in the indigenous and fishing communities, respectively, while their objectives did not significantly vary from the other studies conducted in such settings. The literature search also showed that the HDI and MDS had not been used widely in the Malaysian setting. In summary, the preferred tools for diet quality measurement in Malaysia have been the DDS or FVS in undernutrition or maternal and child health settings and the HEI and its adaptations in settings that focused on chronic diseases. 

Overall, Malaysian diet quality showed scope for dietary improvement across all the populations studied. The HEI and its modified scores in Malaysian studies ranged from 17% to 72%, with a median in the mid 50%. Similarly, DDS scores ranged from 6.38 to 12.69, out of a maximum possible score of 15. This may seem counterintuitive given that Malaysia is well-known as an affordable food haven, a melting pot of rich multi-ethnic culinary traditions. However, Malaysia has been ranked 43rd of 133 countries in the Global Food Security Index (GFSI) [68] and has experienced increased Westernization of its urban diets [3,69]. Eating healthy also has been found to add to daily dietary costs [44]. Therefore, while Malaysia is known for its tasty cuisine, eating healthy every day may be beyond the affordability of the rural and urban poor. This once again is consistently demonstrated in the positive relationship between diet quality and proxy indicators of income (education, household income, personal income, food security, household food expenditure, daily dietary costs) across many studies included in this review. Existing Malaysian studies have predominantly included low-income households, indigenous communities, or households in rural settings, and therefore this sampling could have accentuated the relationship between income and dietary quality. Thus, nutrition intervention programs for the under-privileged could be tailored to the target populations based on their age and ability. Such interventions could include a mixed supply of healthy foods, subsidies for healthier food purchases, nutrition education, kitchen garden establishment, and poverty eradication activities, as required.

Lower diet quality scores in Malaysian studies are seen among school and university students from urban settings and adults from indigenous communities or rural settings. Pregnant women and women of childbearing age also show poor diet quality. This is of concern given that early influences during critical periods of prenatal and postnatal development result in epigenetic changes that impact health and behavioral outcomes of the new-born and that are carried on into adulthood and future generations [70,71,72].

Associations between age and diet quality were inconsistent among the studies reviewed. While two of the reviewed studies [44,57] showed that diet quality improved with age, Leiu et al. [48] and Nohan et al. [49] showed a negative association between age and diet quality. It is important to note that participant age showed a marked spread in only one [44,48,49,57] of the four studies that reported an association between age and diet quality. The food groups found to be deficient in these studies were fruits, vegetables, dairy, and legumes, while excessive intakes of meat, salt, sugar, and fat were also documented. While it is difficult to compare these smaller individual studies to the more extensive national data, it should be noted that two distinct dietary patterns were associated with younger age in the national surveys [3]. Younger adults in Malaysian national surveys were more likely to be associated with two major dietary patterns (i) “Western” (fast-food, carbonated drinks, confectionery, condiments, and sauces) and (ii) “Mixed” (breakfast cereals, fruits, vegetables, dairy, and legumes). These dietary patterns were also associated with urban residence and higher incomes. The majority of the participants in the four studies of interest in this review (approximately 60–100% of respondents) could be classified as coming from low-income households with a monthly household income of less than RM 3500 [73] and were from urban/semi-urban settings (except Chua et al. [57]). Thus, it is unclear whether a dichotomous preference for either a more high-fat-salt-sugar laden “western” pattern or a more prudent “mixed” pattern among the younger respondents in the studies could explain the inconsistency in the association between age and diet quality.

Two studies included in this review showed women to have better diet quality than men. This phenomenon has been noted globally [74] and within Malaysia [3] and is driven by women’s preferences for a healthier lifestyle. More frequently, in Malaysian national data, men adhered to fast food and meat-based dietary patterns compared to women [3]. Ethnicity did not show a consistent relationship with diet quality in the studies we reviewed. This could be of interest to future investigations that appropriately focus their study on that subject.

The associations demonstrated in these studies support the utility of the diet quality indicators used in Malaysia for maternal and child health and NCD risk evaluation. Diet quality assessed using the DDS was shown to be associated with appropriate body weight for age in children, and higher FVS scores were associated with higher energy intake in the study of mother and child dyads. Poorer diet quality indicated by lower HEI/modified HEI scores was associated with being overweight, visceral obesity, higher postprandial glycemia, hypertension, and breast cancer risk in adults. This provides validation for using the HEI or its modified versions to assess unhealthy diets related to chronic diseases in Malaysian adults. In -line with these findings, good dietary quality with better DQI scores also indicated better skeletal mass in the elderly. However, HEI scores were not associated with cardiovascular risks in Malaysian adolescents, despite 35% of the respondents showing at least one metabolic risk. Thus, the FVS and DDS may be useful for the rapid screening of diet quality in studies relating to maternal and child health, while the HEI and its modified versions are useful for studying diet-chronic disease relationships among Malaysian adults. However, the utility of these indices among Malaysian adolescents requires further investigation. It must be noted that the M-HEI used in a few of the included studies evaluates conformance in seven food groups (grains and cereals; vegetables; fruits; meat, poultry and eggs; fish and seafoods; legumes; and milk and dairy products) and two nutrients (fat and sodium) with Malaysian Dietary Guidelines [75]. This iteration of the HEI does not include the evaluation of saturated fat intake, as palm oil is a common cooking oil in Malaysia, and saturated fat intake will not sufficiently differentiate the participants.

This review showed that most diet quality studies in Malaysia included lower-income households from urban, semi-urban, and rural settings predominantly in the Klang Valley, including Selangor and Kuala Lumpur. There were also some data from Terengganu, Negeri Sembilan, and Kelantan. Given that food choice is a combination of affordability, availability, convenience, and conditioned personal preference, concerted efforts should be made toward nationwide representation to better evaluate the influence of sociodemographic factors on diet quality in this multi-ethnic country. Various versions of the HEI have been used in Malaysia. It is crucial to evaluate the agreement between these versions and flag distinctions, if any. Associations between dietary quality for the population and the food environment, including proximity to grocery stores, restaurants, and eateries, would provide the information required for policy formulation. This would be especially important, given the high frequency of eating out in Malaysia [76]. Finally, prospective studies should be undertaken to validate the ability of these indicators to predict chronic disease risk in this population, as the existing evidence is predominantly cross-sectional.

## 5. Conclusions

In this scoping review, we reviewed studies concerned with Malaysian diet quality. The studies revealed an overall scope for improvement in diet quality for the population. Additionally, we demonstrated the validity of food variety-based and nutrient-based scores for specific settings and objectives in the Malaysian context. We also outlined the research gaps and scope for future investigations in this area.

## Figures and Tables

**Table 1:** Category of diet quality indices.

Category	Example	Range of Scores
Nutrient-based indicators	Nutrient adequacy ratios (NARs)	0–100
	Mean adequacy ratio (MAR)	0–100
Food/food group-based diversity indicators	Diet diversity score (DDS)	Variable
Combination indexes	Healthy Eating Index (HEI)	0–100
	Alternate Healthy Eating Index (AHEI) 2010	0–110
	WHO Healthy Diet Indicator (HDI)	0–7
	Diet Quality Index (DQI)	0–100
	Diet Quality Indicator International (DQI-I)	0–100
	Mediterranean Diet Score (MDS)	0–14

**Table 2:** Summary of the study populations.

Population		Number of Studies	Ref.
Multi-ethnic	Children	1	[38]
	Adolescents	2	[39,40]
	Young adults	2	[41,42]
	Adults	6	[43,44,45,46,47]
	Older adults	2	[48,49]
Malay	Adult women	1	[50]
	Mother with at least 1 child	2	[51,52,53,54]
Indian	Women	1	[55]
	Mother with at least 1 child	1	[51]
OA	Children	2	[56,57]
	Adults	1	[58]
	Women	1	[21,22]
	Mother with at least 1 child	1	[59]
Specific groups	Adults with diabetes/prediabetes	3	[60,61,62]
	Men with intellectual disability	1	[63]
	Women with breast cancer	2	[64,65]
	Pregnant women	1	[54]

**Table 3:** Demographic factors associated with diet quality.

	*n*	Children	Adolescents	Young Adults	All Adults	Men	Women	Older Adults	Ref.
Age	4	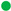			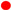 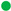			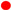	[43,44,49,57]
Higher education	2				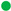			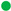	[49,60]
Married	1						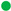		[21,22]
Maternal years of schooling	1	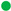							[57]
Malay ethnicity	3		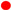				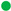	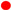	[40,46,49]
Male	1		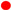						[40]
Female	3		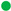		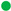 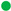				[39,43,61]
Not working	1				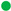				[60]
Household income	2				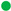		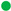		[21,22,60]
Personal income	3				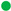		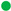	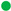	[46,49,60]
Non-Jah Hut OA subtribe	1	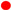							[57]

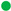
 Positive association; 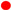 Negative association. Classification was based on multivariate analysis or bivariate analysis.

**Table 4:** Nutritional factors associated with overall diet quality.

	*n*	Children	Adolescents	Young Adults	All Adults	Men	Women	Older Adults	Ref.
Food insecurity	6					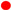	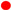 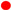 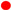 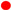 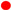		[21,22,47,50,51,52,53,54,55]
Nutritional knowledge	1			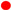					[41]
Eating out	1						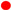		[46]
Food neophobia	1			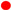					[41]
Sensory	1			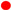					[41]
Household food expenditure	1				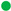				[43]
Higher energy-adjusted daily dietary cost	1				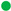				[44]
Healthy lunch (RD4U© meal)	1				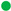				[66]
Availability of healthy foods	1		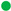						[40]
Healthy eating self-efficacy	1		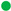						[40]
Energy intake	1	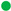					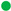		[59]
Higher adjusted carbohydrate intake	1				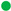				[44]
Higher adjusted fat intake	2				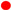		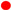		[21,22,44]

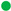
 Positive association; 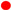 Negative association. Classification was based on multivariate analysis or bivariate analysis.

**Table 5:** Association between overall diet quality and anthropometric measures.

	*n*	Children	Adolescents	Young Adults	All Adults	Men	Women	Older Adults	Ref.
BMI/overweight and obese	3			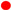	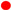		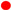		[42,60,64]
Dual-burden malnutrition (OWM/UWC)	1	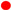					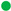		[59]
WC/WHR	2				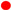			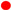	[49,60]
Visceral fat	1							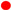	[49]
Body fat	1							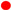	[49]
Skeletal muscle	1							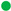	[49]
MUAC	1							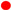	[49]
Handgrip strength	1							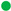	[49]

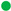
 Positive association; 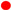 Negative association. Classification was based on multivariate analysis or bivariate analysis. BMI = body mass index; OWM = overweight mother; UWC = underweight child; WC = waist circumference; WHR = waist-hip ratio; MUAC = mid-upper arm circumference.

**Table 6:** Association between overall diet quality and clinical outcomes.

	*n*	Children	Adolescents	Young Adults	All Adults	Men	Women	Older Adults	Ref.
Hypertension	1							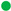	[49]
Serum Hb	1						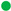		[64]
Excessive GWG	1						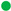		[54]
2-HPP level (pre-diabetes)	1				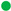				[40]
Pre and post-menopausal breast cancer risk	1						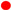		[65]
Insulin treatment (diabetes)	1				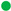				[60]

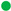
 Positive association; 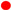 Negative association. Classification was based on multivariate analysis or bivariate analysis. Hb = hemoglobin; GWG = gestational weight gain; 2-HPP = 2-h post-prandial.

## Data Availability

Data sharing not applicable.

## Supplementary Materials

The following are available online at https://www.mdpi.com/article/10.3390/nu13041380/s1, Table S1: Search strategy in Ovid Medline, Table S2: Inclusion and exclusion criteria, Table S3: Summary of all included studies, Table S4: Description of diet quality measures reported in Malaysia.
