# Supplementary material for: Diet Quality of Malaysians across Lifespan: A Scoping Review of Evidence in a Multi-Ethnic Population
Source: Nutrients. 2021 Apr 20;13(4):1380. doi: 10.3390/nu13041380 (PMC8074191; doi:10.3390/nu13041380)
Supplement: Supplementary file 1 [file nutrients-13-01380-s001.zip › nutrients-1146065-supplementary.pdf]

Table S1. Search strategy in Ovid Medline

#	Keyword	Results
1	diet quality.mp.	3459
2	diet quality index.mp.	327
3	healthy eating index.mp.	25
4	HEI.mp.	1170
5	food variety.mp.	200
6	diet variety.mp.	60
7	diet diversity.mp.	197
8	diet diversity score.mp.	29
9	Malaysia.mp. or Malaysia/	18880
10	1 or 2 or 3 or 4 or 5 or 6 or 7 or 8	4571
11	9 and 10	21

*As of 8th February 2021. Comparable strategy was used in other databases.

Table S2. Inclusion and exclusion criteria

Inclusion	Exclusion
• Empirical research done to assess diet quality among Malaysians ***OR***<br>• Empirical research done to assess predictors of diet quality among Malaysians ***OR***<br>• Empirical research done to assess the association between diet quality and an outcome (general well-being, quality of life or any specific clinical outcome) in Malaysians	• Non- peer-reviewed publications eg. conference abstracts, monographs, books or book chapters<br>• Review articles (eg. narrative, scoping or systematic reviews)<br>• Study protocols<br>• Studies conducted in vitro or in vivo<br>• No specific mention/discussion of diet quality/diversity<br>• Articles on diet quality tool development or validation<br>• Not conducted in Malaysia / among Malaysians

Table S3. Summary of all included studies

	Study	Design	Population	Setting	Sample size	Mean age (years)	Diet quality measure	Main findings
1	Shariff et al (2002) [39]	CS	OA children (3-6 years)	OA households	64	N/A	Adapted DQI	• 68.7% of the children had poor diet quality (73.8% of males and 59.1% of females). None had excellent diet quality.<br>• Diet quality of the children decreased as household food insecurity worsened. Poor diet quality seen in 81% of individual food insecure, 77.8% households with child hunger, 63.8% of household insecure and in 50% of food secured household.
2	Shariff et al (2005) [50]	CS	Malay and Indian mothers (>20 years) with at least one child aged 1-6 years	Rural households	200 (Malays = 140 & Indians = 60)	33.9±7.6	FVS	• The mean for FVS was significantly higher between food-secure and all food-insecure groups, and between household food-insecure and child hunger groups.<br>• Overweight and abdominal adiposity among the women were associated with several independent variables including lower FVS.
3	Saibul et al (2009) [65]	CS	OA Mothers (18 to 55 years) with at least 1 child aged 2 to 9 years	OA households	182 mothers & 284 children	Mothers = 30.8±7.8; Children = 4.5±2.6	FVS	• Mean FVS was similar for women (7.0±2.1) and children (6.9±1.9).<br>• Dual burden households (overweight mother/underweight child) were associated with FVS of children (OR: 0.71, 95% CI: 0.51-0.95) and FVS of women (OR: 1.39, 95% CI: 1.02- 1.89). The FVS of children (OR: 0.49, 95% CI: 0.25-0.89) and women (OR: 1.92, 95% CI: 1.64-2.77) remained significant even when dual burden households were compared to only households with normal weight mother/normal weight child.
4	Karppaya et al (2010) [45]	CS	OA adults (Che Wong tribe) (18 years and above)	OA households	57	Men: 39.9 ± 17.1; Women: 33.7 ± 16.1	DDS	• Mean DDS was 9.47 ± 4.15; men and women had similar scores (9.48 ± 3.70 vs 9.46 ± 4.63).<br>• There was significant correlation between waist circumference and household income (r=0.36, p<0.01), but the association was only significant in women (r=0.50, p<0.01)

5	Mohamadpour et al (2012) [47]	CS	Indian adult women (19-49 years)	Rural households	169	Food secure: 37.12±9.09; Household food insecurity: 39.52±7.30; Individual food insecurity: 40.61±7.37; Child hunger: 38.74±6.77	DDS	• Women who reported food security had significantly higher mean DDS (11.60±4.13) than child hunger group (9.23±3.36).<br>• DDS shown to be a significant protective factor against health risks even after adjusting for other variables.
6	Sulaiman et al (2012) [48]	CS	Malay adult women (19-49 years)	Rural & urban households	301	N/A	DDS	• Mean DDS of food secure household (11.61 ± 3.70) was significantly higher than moderate household food insecurity (8.79 ± 3.31) & severe food insecurity (8.50 ± 3.51) ($p<0.01$)
7	Chua et al (2012) [40]	CS	OA children (Temuan, Jah Hut &Che Wong sub-tribes) (1-6 years)	OA households	216	41.4 months	DDS	• Mean DDS of children was 6.38 of the possible 15. The DDS of most children were in the lower (56.0%) and middle (36.1%) tertiles.<br>• Higher DDS of children significantly related to older age, non-Jahut sub-tribe, longer maternal years of schooling. DDS also positively related to weight-for-age z-scores and height-for-age z-scores.
8	Badari et al (2012) [41]	CS	Adults (20-65 years)	Urban community	285	39.4 ± 12.3	DDS, FVS	• The overall mean of food variety scores was 164.1 ± 93 with highest mean intake for fish, poultry, meat, and legume groups (35.6 ± 28). 33.7% of the households had low FVS while 34.0% had medium FVS. Only 32.3% of households had higher FVS.<br>• Age and sex of respondents were significantly different with mean household FVS.<br>• DDS show that the weekly mean intake of respondents was 6.0 ± 0.4, with most of them having a high DDS (88.4%).

								• Significant differences in DDS of milk products group between household incomes and (p<0.01).<br>• Household food expenditure had a significant and positive linear relationship with DDS.
9	Shahril et al (2012) [59]	CC	Women with and without breast cancer (18-80 years)	Tertiary urban hospitals	764 (382 cases & 382 controls)	49.8 ± 10.6 (case); 49.7 ± 11.2 (control)	HEI-2005	• Total HEI-2005 score was lower among premenopausal breast cancer cases than healthy controls (64.8±9.7 vs 67.9±8.8; p< 0.001). Similar observation seen among postmenopausal breast cancer cases (64.3+9.3 vs 67.9+8.8; p<0.001).<br>• Significant reduction in the risk of breast cancer, with a higher HEI-2005 total score among premenopausal women (OR Q1 vs. Q4= 0.34, 95%CI; 0.15–0.76) and postmenopausal women (OR Q1 vs.Q4= 0.20, 95% CI;0.06–0.63).<br>• HEI-2005 has a sensitivity of 56–60%, a specificity of 55–60%, and a PPV and NPV of57–58%, which indicates a moderate ability to predict the risk of breast cancer according to menopausal status.<br>• The breast cancer incidence observed poorly agrees with risk outcomes from HEI-2005 as shown by low-j statistics(j= 0.15).
10	Karupaiah et al (2013) [46]	CS	Adult women (19-65 years)	Urban community	128	N/A	Adapted HEI (1995)	• 55.5% of women were identified as the "need improvement" category (HEI score = 73.6 ± 5.7) whilst the remaining 44.5 % achieved 'good' diet quality (HEI score = 86.4 ± 3.4)<br>• Total HEI score was significantly different within ethnicity (Indians = 75.7 ± 8.1 < Malays = 80.5 ± 7.4 < Chinese = 80.1 ± 8.1).<br>• Better total HEI scores were associated with larger monthly incomes (p = 0.159, P = 0.048).<br>• Diet quality for participants consuming home prepared meals was 'good' (17.2 %, 82.0 ± 8.0) compared to those eating one to two meals outside (68.8 %, 78.8 ± 7.8) or all meals outside (14.0 %, 78.3 ± 8.9); consuming meals outside was negatively correlated with HEI score (rs = -0.149; p= 0.046) with

								a decline in vegetable intake being a major factor (rs= -0.320,p<0.001).<br>• Diet quality of women with BMI<18.5 was related to a poorer HEI score (71.7, p= 0.016).
11	Teng et al (2013) [44]	RCT	Adult men (50-70 years)	Urban community	25	58.8 years	Adapted HEI (2005)	• Trial compared effect of 12-week Fasting Calorie Restriction (FCR) vs control group. Total HEI score improved in both groups, with better improvement in FCR group (60.1±8.5 to 68.6±3.7) compared to controls (61.7±8.1 to 64.2±6.).<br>• Significant reduction in food variability in FCR (9.5±0.9 to 8.1±1.1; p<0001).<br>• A significant main effect of HEI score seen for fat, saturated fat and cholesterol scores, a significant time effect of HEI score seen for fruit and fat.<br>• Overall HEI still categorized as "need improvement".
12	Shariff et al (2014) [49]	CS	Adult women in reproductive age (19-49 years)	Rural & urban households	625	Food secure: 38.1±7.1; Household food insecurity: 38.0±7.1; Individual food insecurity: 38.4±7.2 ; Child hunger: 37.7±7.2	DDS	• Food secure women (10.7±4.36) had significantly higher DDS than women from individual food insecure (9.47±3.66) and child hunger households (9.07±3.70).
13	Tiew et al (2014) [60]	CS	Adults with diabetes (18 years and above)	Tertiary hospital	113	54.1±10.3	DDS	• Mean Food Group Score (FGS) and Serving Score (SS) were 4.12±0.79 and 12.75±3.50 respectively.<br>• 34.5% of the patients had perfect FGS while only 1.8% had perfect SS.<br>• Lower education, lower personal income, working, non-insulin, overweight and obese subjects had significantly lower FGS than their counterparts whereas lower education, lower waist-to-hip ratio,

								overweight and obese subjects had significantly lower SS than their counterparts.
14	Ihab et al 2015); Ihab et al (2013); Ihab et al (2012) [52-54]	CS	Malay mothers (18 to 55 years) with at least 1 child aged 2 to 12 years	Rural households	223	42.2 ± 6.4	DDS	• Mean DDS in respondents from food secure households is significantly higher than those from food insecure households (12.69 vs 7.63; p<0.001).<br>• Meat and meat products, fruits and milk and dairy products were associated with food security status.<br>• No significant difference in DDS scores between overweight-mother/underweight-child and normal-weight-mother/normal-weight-child mother-child pairings.
15	Rezali et al (2015) [64]	CS	Adolescents (13-16 years)	Secondary school	373	14.3 ± 1.2	M-HEI	• The composite M-HEI score between 16.7% - 65.6%, with a mean of 37.9 ± 9.1%. Poorer diet quality in males than females (34.2 ± 8.2% vs 39.9 ± 9.0%; p<0.05). Malay adolescents had poorer diet quality than Indians (36.9 ± 8.7% vs 41.3 ± 10.0%, p<0.05).<br>• Majority of the adolescents (80.7%) were at risk of poor diet quality while the remaining 19.3% had a low risk of poor diet quality.<br>• Significant weak correlation between age and diet quality of the adolescents (r = 0.123; p< 0.05).
16	Shu et al (2017) [61]	CS	Adults with diabetes (18–65 years)	Tertiary hospital	155	53.0±9.4	Adapted HEI (2005)	• Mean total HEI score is 71.53±10.16.<br>• Diet quality of the subjects was unsatisfactory especially for vegetables, fruits, fish and legumes as well as from the milk and dairy products group.<br>• Majority of them (76.8%) needing to improve their diet quality and only 1.9% had good diet quality.
17	Suhaimi et al (2017) [66]	CS	Young adults (18–25 years)	Universities	400	22	FVS	• 95.25% of the students had a low level of food variety in every food groups.<br>• Respondents who chose food based on sensory motive and neophobic had 1.54 and 5.55 likelihood to have low level food variety, respectively.<br>• Those with high level of nutritional knowledge were 5.40 more likely to have low level of food variety.

18	Pondor et al (2017) [42]	CS	Adults (18-64 years)	Urban community	450	37.6 ± 11.0	M-HEI	• Significant positive association between higher energy adjusted daily dietary cost (DDC) and higher mean M-HEI scores.<br>• Significant positive associations between higher energy adjusted DDC and higher component M-HEI scores for cereal products, vegetables, and fruits.<br>• Older participants (age group ≥ 60years) had higher mean M-HEI (70.22 ± 11.95) compared to 20–29 years old (59.70 ± 9.90; p = 0.04).
19	Chua et al (2018) [38]	CS	Children (7 to 12 years)	Rural households	100	Median = 10 (IQR=4)	Adapted HEI (1995)	• The diet quality of subjects was poor, with mean total HEI score of 50.45±5.27 out of 100 points.<br>• Poor compliance with dietary guidelines shown by low median scores of vegetable (0.83), fruit (0.00) and dairy (0.00) components.
20	Khalib et al (2018) [43]	RCT	Adults (18-59 years)	University & hospital	50	39.4 ±9.7	M-HEI	• Total M-HEI score of the intervention meal (RD4U© meals) was higher than that for the usual lunch meals (61.9±9.2 vs 56.1±11.2) (p<0.001).<br>• Overall diet quality scores indicate both RD4U© and usual lunch meals were in the "need improvement" category.
21	Yap et al (2019) [67]	CC	Young adults (18–25 years)	Universities	294 (150 = normal weight & 144 = obese/overweight)	20.3±1.8	Adapted HEI (1995)	• The total HEI score was significantly lower in OW/OB group (45.4+11.3 vs 51.43+11.61). Lower scores were seen for energy from fat and saturated fat, cholesterol and sodium intake in OW/OB group. OW/OB group achieved a significantly higher component score for cereals and meat food groups. Both groups achieved low component scores for fruits, vegetables, milk, and food variety.
22	Jamil et al (2019) [57]	CS	Men with and without intellectual disability (20-40 years)	Urban community-based rehabilitation	95	Median = 23 (IQR=8)	M-HEI	• Total HEI score was significantly higher in men with intellectual disability (53.6) compared with the participants without intellectual disability (39.7).<br>• Majority of men without intellectual disability (83%) had poor diet quality whilst more than half of

				centres and universities				intellectual disability men (60%) were required to improve their dietary intake.<br>• HEI was not associated with calcaneal speed of sound.
23	Chong et al (2019); Chong et al (2018) [21,22]	CS	OA women (Mah Meri) (19-59 years)	OA households	222	36.5 ± 11.5	M-HEI	• Overall, poor diet quality (mean M-HEI: 45.3 ± 7.5%).<br>• M-HEI scores positively correlated with household income and nutrition knowledge.<br>• Food-secure group was significantly associated with a higher M-HEI score after controlling for age.
24	Yong et al (2019) [54]	LS	Women in the first trimester (10–13th weeks of gestation)	Primary care clinics (Seremban Cohort Study)	480	30.2±4.5	M-HEI	• Lower overall HEI score across the trimesters.<br>• Significant differences in the HEI component score across trimesters, except for fruits.<br>• Higher HEI score for cereals and grains (7.53–8.54), poultry, meat, and egg (7.52–8.55) and sodium (7.04–8.61), but lower HEI score for legumes (1.04–3.14) and milk and milk products (1.96–3.82).<br>• Overweight/obese women had lower total HEI score (51.49–55.40) during pregnancy compared to non-overweight/obese women (53.38–56.50). Overweight/obese women with higher total HEI scores in the second (aOR=1.04, 95% CI=1.01–1.07,p=0.02) and third trimester (aOR=1.04,95% CI=1.01–1.08,p=0.02) were significantly at higher risk for excessive GWG.<br>• In non-overweight/obese women, higher total HEI scores in the second and third trimesters were significantly associated with lower risk of inadequate GWG (aOR=0.97, 95% CI=0.95–0.99,p=0.01) and higher risk of excessive GWG(aOR=1.04, 95% CI=1.01–1.07,p=0.03), respectively.
25	Nohan et al (2020) [56]	CS	Older adults (>60 years)	Urban community	138	68.0 ± 6.0	DQI	• 74.6% of respondents have good diet quality, whilst 25.6% had poor diet quality.<br>• Increasing age, Malay ethnicity, low education attainment, low financial income, hypertension, low skeletal muscle, high body fat, high visceral fat, high

								waist circumference, low MUAC and low handgrip strength were associated with the diet quality.
26	Ng et al (2020) [58]	CS	Women with breast cancer (29 – 71 years)	Hospital	163	50.3	HEI-2015	• The mean total HEI score was 63.86±8.75.<br>• Those who perceived higher score of total HEI-2015 were less likely to experience overweight (Q4 vs Q1: OR=0.36; 95% CI=0.13, 0.99) while reported higher concentration of serum hemoglobin (Q4 vs Q1: OR=0.36; 95% CI=0.13, 0.98; Q3 vs Q1: OR=0.35; 95% CI=0.13, 0.92).<br>• The median scores for adequacy components of total fruit, whole fruit, total vegetables, greens and beans, total protein food, seafood and plant proteins, as well as moderation components of sodium, added sugars and saturated fats achieved the maximum scores of 100%.<br>• Whole Grains, dairy, fatty acids and refined grains scored less than 25% of the maximum.
27	Appanah et al (2020) [63]	CS	Adolescents (13 years)	Secondary school	336	13.5 ± 0.3	M-HEI	• Females had significantly higher mean M-HEI score compared to males (50.3 vs 46.3; p=0.02).<br>• No significant associations were observed among adolescents in the lowest quartile compared to those in the highest quartile of M-HEI score for obesity as well as other cardiometabolic risk factors in both males and females.
28	Siddiqui et al (2020) [62]	CS	Adults with undiagnosed / prediabetes (18 years and above)	University hospital	147	N/A	M-HEI	• Overall, diet quality of study participants was unsatisfactory with the mean score of 58.05±9.07 (need improvement).<br>• Lowest composite score seen in prediabetic group.<br>• Total HEI score was negatively correlated with the 2-HPP levels in pre-diabetic patients (rs=-0.45, p<0.05).<br>• No significant association was revealed between glycemic parameters and total HEI score among other groups.

29	Leiu et al (2020) [55]	CS	Older women (50 years and above)	NGO	214	67.2 ± 6.6	M-HEI	• The mean M-HEI composite score was 66.9 ± 9.9.<br>• No significant association between M-HEI score with vitamin D status.

Footnote: CS = cross-sectional; CC = case-control; RCT = randomized-controlled trial; OA = Orang asli; HEI = Healthy Eating Index; M-HEI = Malaysian Healthy Eating Index; DQI = Diet Quality Index; DDS = Diet Diversity Score; FVS = Food Variety Score; N/A = not available

Table S4. Description of diet quality measures reported in Malaysia

	Measure	Components	Range of score	Diet quality categorization	Studies
Diversity/variety score	DDS	Varied according to studies:<br>1) The number of food groups (from 29 food group representing 7 categories of grains & cereals; meat & meat products; fish & seafoods; fruits; vegetables; milk & dairy products; meat-alternatives; beverages) consumed regularly (daily or ≥2 times per week).	0 – 29	A higher score reflects greater diversity of the diet.	Mohamadpour et al (2012), Shariff et al (2014), Sulaiman et al (2012) [47-49]
		2) Food Group Score (FGS) and Serving Score (SS) adapted from from Kant et al. (1991). The FGS reflected the number of food groups consumed daily from 5 food groups—grains (cereals, tubers & grains); fruits; vegetables; meat (fish, poultry, meat, eggs & legumes); dairy (milk & dairy products). SS reflected the presence of achieving the minimum recommended number of servings for the 5 food groups— four servings daily for grains, and two servings daily each of fruits, vegetables, meat, and dairy.	FGS: 0 – 5<br>SS: 0 – 2	A higher score reflects greater diversity of the diet.	Tiew et al (2014) [60]
		3) The number of food groups consumed within a week, modified from Clausen et al (2005) and based on 6 food groups from Malaysian Food Pyramid (cereals, cereal products & tubers; vegetables; fruits; fish; poultry, meat & legumes; milk & milk products, and fat, oils, sugar & salt).	0 – 6 per food group	≤3: lowest diversity<br>4-5: medium diversity<br>≥6: high diversity	Badari et al (2014) [41]
		4) The number of food groups (from 8 categories - cereals & cereal products; meat & meat products; fish; fruits; vegetables; legumes; milk & dairy products; and beverages) consumed within a week (daily or ≥2 times per week).	0 – 30	A higher score reflects greater diversity of the diet.	Ihab et al 2015), Ihab et al (2013), Ihab et al (2012) [52-54]
		5) The number of food groups (from 37 food groups representing 7 categories of cereals & cereal products; meat & meat products; fish; fruits; vegetables; milk & dairy products, and beverages) within a week.	0 – 37	A higher score reflects greater diversity of the diet.	Karppaya et al (2010) [45]
		6) The number of food groups (from 15 food groups) consumed within a week (daily or ≥3 times per week).	0 - 12	A higher score reflects greater diversity of the diet.	Chua et al (2012) [40]

	FVS	1) Scoring approach adapted from Clausen et al (2005), based on weekly household consumption of 12 categories food groups (116 items) that were consumed at least once week.	0 - 812	<33rd percentiles: low FVS<br>33rd-64th percentiles: medium FVS<br>65th - 100th percentiles: high FVS	Badari et al (2014), Suhaimi et al (2017) [41,66]
		2) Total number of food items in the FFQ (58 possible food items) consumed regularly (daily or 2–3 times a week)	0 - 58	A higher score reflects greater food variety.	Shariff et al (2005) [50]
		3) Total number of food items consumed over a 3-day period, from a possible total of 69 food items (from categories of grains & cereals; fruits; vegetables; milk & dairy products; meat/poultry/egg; fish; and seafood identified from 3-day 24-hour diet recalls.	0 – 69	A higher score reflects greater food variety.	Saibul et al (2009) [65]
Healthy eating index	M-HEI	9 components - 7 food groups (cereals & grains; vegetables; fruits; milk & milk products; poultry, meat & eggs, fishes; and legumes) and 2 nutrient (total fat and sodium) components, adhering to Malaysian Dietary Guidelines / Malaysian Dietary Guidelines for Children and Adolescents	0 – 100	A higher score reflects greater diet quality.	Pondor et al (2017), Leiu et al (2020), Yong et al (2019) [42,54,55]
				≤46%: high risk of diet quality<br>>46%): low risk of poor diet quality	Appanah et al (2020), Rezali et al (2015) [63,64]
				<51: poor diet<br>51 – 80: needs improvement<br>>80: good diet	Khalib et al (2018), Siddiqui et al (2020), Jamil et al (2019), Chong et al (2018), Chong et al (2019) [21,22,43,57,62]
	Adapted HEI (1995)	10 components (grains; vegetables; fruits; dairy; meat; total fat; saturated fat; cholesterol; sodium; and variety) modified to adhere to Malaysian Dietary Guidelines for Children and Adolescents 2014.	0 – 100	<51: poor diet<br>51 – 80: needs improvement<br>>80: good diet	Karupaiah et al (2013), Chua et al (2018), Yap et al (2019) [38,46,67]
	Adapted HEI (2005)	10 components modified to adhere to Malaysian Dietary Guidelines 2010 - of 10 dietary components (grains & cereal products;	0 – 100	<50: poor diet quality	Teng et al (2013), Shu et al (2017) [44,61]

		vegetables; fruits; milk & dairy products; fish, meats & legumes; total fat; saturated fat; cholesterol; sodium; and variety)		51 – 80: needs improvement<br>>80: good diet quality	
	HEI (2015)	13 components adhering to Dietary Guidelines for Americans 2015-2020 - 9 adequacy (total fruits, whole fruits, total vegetables, greens & beans, whole grains, dairy, total protein food, seafood & plant proteins, and fatty acids) and 4 moderation (refined grains, sodium, added sugars and saturated fat) components	0 - 100	A higher score reflects greater diet quality.	Ng et al (2020) [58]
	HEI (2005)	12 components using recommendations of the Dietary Guidelines for Americans 2005 -total fruits, whole fruits, total vegetables, dark green & orange vegetables & legumes, total grains, whole grains, milk, meat & beans, oils, saturated fat, sodium, and calories from solid fat, alcoholic beverages & added sugars	0 – 100	A higher score reflects greater diet quality.	Shahril et al (2012) [59]
Diet quality index	DQI	12 components using The Malaysian Dietary Guidelines & Malaysian Food Pyramid as guides - cereal, cereal products & tubers, wholegrain cereals, fruits, vegetables, milk & dairy products, legumes & their products, fish, poultry, meat & egg, high-fat protein foods, fat-rich foods, salt rich foods, and sugar-rich foods.	0 – 60	< 29.9: at risk of poor diet quality<br>>30: low risk of poor diet quality	Nohan et al (2020) [56]
	Adapted DQI	5 food groups adapted from DQI (Patterson et al) according to Malaysian Food Guide Pyramid far children– cereal, cereal products & tubers, vegetables, fruits, milk & dairy products, and fish, poultry, meat & legumes.	0 – 10	0 – 4: poor diet quality<br>5 – 9: fair diet quality<br>10: excellent	Shariff et al (2002) [39]
